# Uncovering the impact of randomness in HIV hotspot formation: A mathematical modeling study

**DOI:** 10.1371/journal.pcbi.1013178

**Published:** 2025-06-16

**Authors:** Nao Yamamoto, Daniel T. Citron, Samuel M. Mwalili, Duncan K. Gathungu, Diego F. Cuadros, Anna Bershteyn

**Affiliations:** 1 Department of Population Health, New York University Grossman School of Medicine, New York, New York, United States of America; 2 Strathmore University, Ole Sangale Road, Nairobi, Kenya; 3 Jomo Kenyatta University of Agriculture and Technology, Nairobi, Kenya; 4 Digital Epidemiology Laboratory, Digital Futures, University of Cincinnati, Cincinnati, Ohio, United States of America; University of Melbourne, AUSTRALIA

## Abstract

**Background:**

HIV hotspots, regions with higher prevalence than surrounding areas, are observed across Africa, yet their formation and persistence mechanisms remain poorly understood. We hypothesized that random fluctuations during the early stages of the HIV epidemic (1978–1982), amplified by positive feedback between HIV incidence and prevalence, play a critical role in hotspot formation and persistence. To explore this, we applied a network-based HIV transmission model, focusing on randomness in the spatial structure of the epidemic.

**Methods:**

We adapted a previously validated agent-based network HIV transmission model, EMOD-HIV, to simulate HIV spread in western Kenya communities. The model includes demographics, age-structured social networks, and HIV transmission, prevention, and treatment. We simulated 250 identical communities, introducing stochastic fluctuations in network structure and case importation. Outliers were defined as communities with prevalence > 1.5x the median, and persistence as meeting these criteria for >70% of 1980–2050. We systematically varied community size (1,000–10,000), importation timing (1978–1982), and importation patterns (spread over 1, 3, or 5 years), and calculated the proportion of outliers and persistent outliers.

**Results:**

HIV prevalence outliers were more common in smaller communities: in 1990, 25.3% (uncertainty interval: 22.3%–28.2%) of 1,000-person communities vs. 9.1% (uncertainty interval: 6.9%–11.4%) of 10,000-person communities. By 2050, 21.6% of 1,000-person communities were persistent outliers, compared to none in larger communities. Autocorrelation of HIV prevalence was high (Pearson’s correlation coefficient 0.801 [95% CI: 0.796–0.806] for 1,000-person communities), reflecting feedback that amplified early fluctuations.

**Conclusions:**

Early random fluctuations contribute to the emergence and persistence of prevalence outliers, especially in smaller communities. Recognizing the role of randomness in prevalence outlier formation in these settings is crucial for refining HIV control strategies, as traditional methods may overlook these areas. Adaptive surveillance systems can enhance detection and intervention efforts for HIV and future pandemics.

## Author summary

In this study, we explored how random events early in the HIV epidemic may have contributed to the formation of HIV prevalence outliers. Using a simulation model of HIV spread in communities in western Kenya, we introduced random variations in the initial spread of the disease and examined how these fluctuations affected infection patterns over time. We found that community-level prevalence exceeding 1.5 times the median could emerge through random variations in an appreciable fraction of small communities, and that outliers were more likely to persist over time in such settings. In contrast, larger communities appeared to need additional factors beyond random chance for outliers to form. Our findings suggest that random fluctuations can influence the development of HIV prevalence outliers, especially in smaller communities. Recognizing this could enhance public health strategies by encouraging more detailed monitoring of HIV in vulnerable areas. By understanding the role of randomness, we can refine approaches to detecting and controlling the spread of HIV and other infectious diseases in the future.

## Introduction

Four decades into the HIV/AIDS pandemic, there remains an incomplete understanding of why certain communities are disproportionately affected by the virus [1–2]. Despite extensive research, two-thirds of people living with HIV (PLHIV) reside in Africa [3], with the eastern and southern regions bearing the brunt of the epidemic. At the national level, the Kingdom of Eswatini has the world’s highest HIV prevalence, affecting 27% of adults [4]. Sub-nationally, similar high prevalence rates are found along the Lake Victoria coast in Eastern Africa [5], particularly within fishing communities [6–8], and South Africa’s KwaZulu-Natal Province [9]. At finer, community-level scales, HIV prevalence [10–13] and incidence [14] exhibit significant variation, sometimes differing by several-fold across neighborhoods just a few kilometers apart. In KwaZulu-Natal, door-to-door surveys have revealed that HIV prevalence can differ more than three-fold between isigodis (neighborhoods) located just 1–10 kilometers apart [15–17]. While individuals often select sexual partners within their own isigodi [10,18]. reinforcing localized transmission dynamics, evidence also suggests that migration plays a role in shaping these patterns, as PLHIV may be more likely to relocate to high-prevalence communities [19].

These stark differences in HIV prevalence, observable across various spatial scales, resemble fractal patterns—mathematical structures that exhibit self-similarity, meaning similar patterns recur at different levels of magnification [20–23]. Fractals frequently occur in nature [24,25], suggesting that further study of HIV’s spatial variation could yield new insights into the epidemic’s dynamics. A range of behavioral and biological mechanisms have been proposed to explain this variability [26,27], including the formation of “hotspots”—areas with anomalously high HIV prevalence compared to neighboring regions [28]. In some cases, hotspots are linked to behaviors and situations that increase HIV risk, such as transactional sex [29,11], cultural practices like polygamy [30], or occupations involving extended separation from partners, such as mining [31,32], long-distance trucking [33], or agricultural work [34]. Biologically, hotspots often coincide with areas where male circumcision rates are lower [35,36], although recent public health initiatives have increased circumcision coverage in many communities [36]. Additionally, hotspots frequently overlap with areas of high prevalence of other sexually transmitted infections (STIs) and reproductive tract infections (e.g., vaginal dysbiosis) [37]. However, the causal associations between these infections and HIV are often confounded by shared behavioral and biological risk factors.

Despite advances in understanding the drivers of the HIV epidemic, many community-level hotspots remain unexplained. In KwaZulu-Natal, the observed three-fold differences in HIV prevalence did not align with measurable differences in behavioral or biological risk factors [15–17]. Proximity to major roads was associated with some hotspots [16], suggesting that increased mobility, such as the movement of migrant workers and truck drivers, could play a role in the spatial distribution of HIV. In rural Kenya’s Nyanza counties, cultural factors, such as stigma against individuals perceived to have HIV/AIDS, often attributed to beliefs in witchcraft or family curses, may also contribute to the classification of certain geographical areas as hotspots [38–41].

The phenomenon of “patchy” and seemingly random epidemic hotspots is not unique to HIV; it has been observed across various infectious diseases and is often associated with stochastic processes in finite populations. Such fluctuations tend to be more pronounced in smaller populations [42,43]. Given HIV’s chronic nature, we hypothesize that stochastic fluctuations in early HIV case introductions may have led to the sustained patchy patterns observed today [44]. Despite this broader phenomenon, there is no universally accepted threshold for defining HIV “hotspots.” Some definitions use absolute prevalence cutoffs, while others compare prevalence within a region to its surroundings. For instance, hotspots have been defined as communities where HIV prevalence is at least twice that of neighboring areas, as observed in fishing communities around Lake Victoria [28], while others report even greater disparities, such as a 2.5-fold difference in prevalence between high-burden fishing communities and surrounding regions [45]. However, a strict threshold may not always capture the full spectrum of spatial heterogeneity, particularly in settings with fine-scale prevalence variations. Given this variability, we define HIV prevalence outliers based on a relative threshold (1.5x the median prevalence across simulated communities) and conduct sensitivity analyses to examine how different threshold choices influence our findings.

This study aims to assess the extent to which randomness in HIV case introductions and spread through sexual networks contributes to the formation of high-prevalence communities. We employed a stochastic, network-based HIV transmission model to simulate identical communities, varying only in random perturbations to sexual networks and initial case introductions while holding average behavioral and biological risk parameters constant. By systematically varying population size, intensity, and timing of case introduction, we examined conditions under the formation of communities with high HIV prevalence could arise under random fluctuations in initial seeding events. Through this approach, we establish a baseline for understanding the degree of variability that can arise due to chance alone. This baseline can be useful in assessing which deviations in prevalence are unusual for a community’s size and intensity of case importation, and therefore potentially indicative of epidemiological risk factors for HIV transmission. We further assessed the sensitivity of the observed patterns to alternative methods of outlier detection and thresholds that define an outlier. This work represents, to our knowledge, the first study exploring when and to what extent HIV prevalence outliers can arise purely by chance, independent of underlying behavioral or biological risk factors. By recognizing the role of stochastic processes, public health strategies can better account for the possibility of randomly emerging high-prevalence areas.

## Results

### Overall prevalence

Across the 93,750 simulations used in the analysis, mean HIV prevalence was 20.1%, with a standard deviation (SD) of 5.8%, in 2018, which was within the uncertainty range of HIV prevalence in Kisumu County estimated in the same year via population-based surveillance (17.5%, 95% CI: 13.6-21.3%). Mean HIV prevalence differed by initial community (i.e., social network) size (S1 Table), with prevalence in 2018 at 17.2% (uncertainty interval: 17.0–17.3%) for communities with an initial population of 1,000 and 21.4% (uncertainty interval: 21.3–21.4%) for communities with an initial population of 10,000.

### Population size

**Fig 1:**
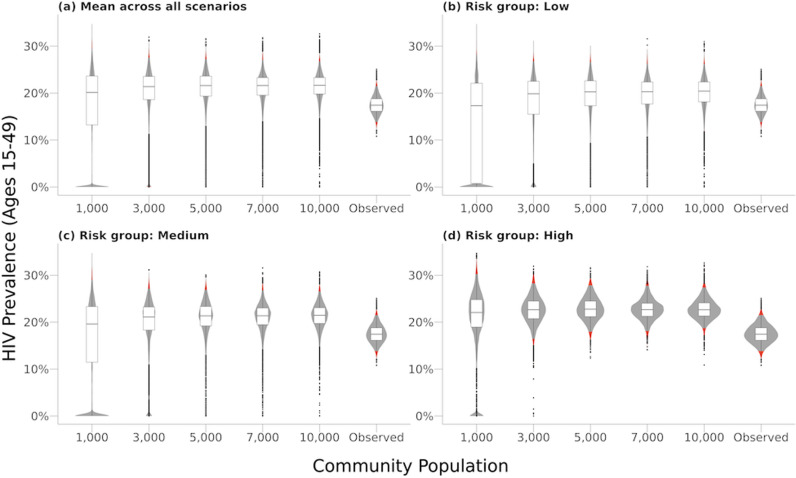
Violin plots of HIV prevalence in 2018 across different initial population sizes compared to the empirical HIV prevalence in Kisumu County. The distribution of simulated HIV prevalence in 2018 for various initial population sizes (1,000, 3,000, 5,000, 7,000, and 10,000 individuals) compared to empirical HIV prevalence (“Observed”) in Kisumu County in 2018. The violin plots show the distribution across 250 simulations for each community size, with boxplots indicating the median and interquartile range (IQR). Panel (a) shows all risk groups combined, and (b) presents results for low-risk groups, (c) for medium-risk groups, and (d) for high-risk groups.

Communities with smaller initial population sizes exhibited systematically larger variance in HIV prevalence across all timepoints. Fig 1 presents violin plots showing the distribution of simulated HIV prevalence in 2018 for communities with varying initial population sizes (1,000; 3,000; 5,000; 7,000; and 10,000 individuals). These plots highlight the variability in prevalence across simulations and compare them to the observed empirical HIV prevalence in Kisumu County for the same year. Notably, the spread of HIV prevalence is broader in smaller populations, especially in communities with an initial population size of 1,000. This increased variability underscores how random fluctuations in transmission dynamics can lead to substantial differences in prevalence within smaller populations.

**Fig 2:**
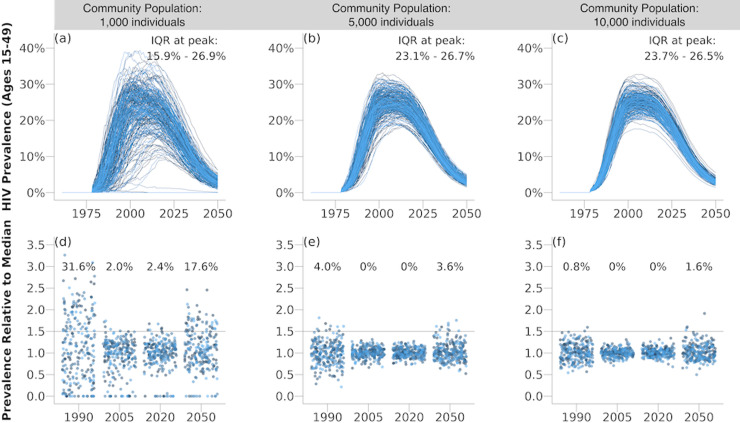
Impact of random fluctuations in HIV case introduction and spread on the formation of HIV prevalence outliers across different population sizes. (a-c) Ensembles of 250 simulation trajectories for population sizes of 1,000, 5,000, and 10,000 individuals, respectively. These curves illustrate the variability in epidemic trajectories due to random perturbations. The interquartile range (IQR) of peak HIV prevalence is noted for each population size. (d-f) Scatter plots showing relative HIV prevalence over time for each population size (1,000, 5,000, and 10,000 individuals). Each point represents a simulation at a specific time point, with the horizontal line indicating a relative prevalence threshold of 1.5, used to define a prevalence outlier as having a prevalence >1.5 times the median prevalence. The percentages above the scatter plots indicate the proportion of simulations that resulted in prevalence outliers for each year.

All simulated epidemics peaked in the mid-2000’s and declined by 2050, reflecting HIV treatment and prevention scale-up. Although absolute variance in HIV prevalence was highest when epidemics peaked (Fig 2), the relative variance (i.e., variability in prevalence relative to the mean prevalence) was highest during the early HIV epidemic (e.g., 1990) and as the HIV epidemic became increasingly controlled (e.g., 2050). In panels (d-f) in Fig 2, each scatter plot represents the relative HIV prevalence at various time points, with the horizontal line marking the threshold of 1.5 times the median prevalence used to define HIV prevalence outliers. A higher proportion of simulations for smaller populations cross this threshold, particularly in the early years of the epidemic. This observation suggests that randomness in early transmission events, in the absence of additional importations, results in greater divergence in prevalence estimates across simulations, especially in smaller communities where random variations have a more significant impact. As population size increases, fewer simulations exceed the threshold for prevalence outliers, indicating that the effects of stochasticity are diminished in larger populations. Overall, Fig 2 demonstrates that random fluctuations in case introduction and transmission dynamics contribute to the emergence of prevalence outliers, particularly in smaller populations. In larger populations (≥5,000), the proportion of simulations exceeding the 1.5x threshold was low (<5%), suggesting a reduced influence of stochasticity alone.

**Fig 3:**
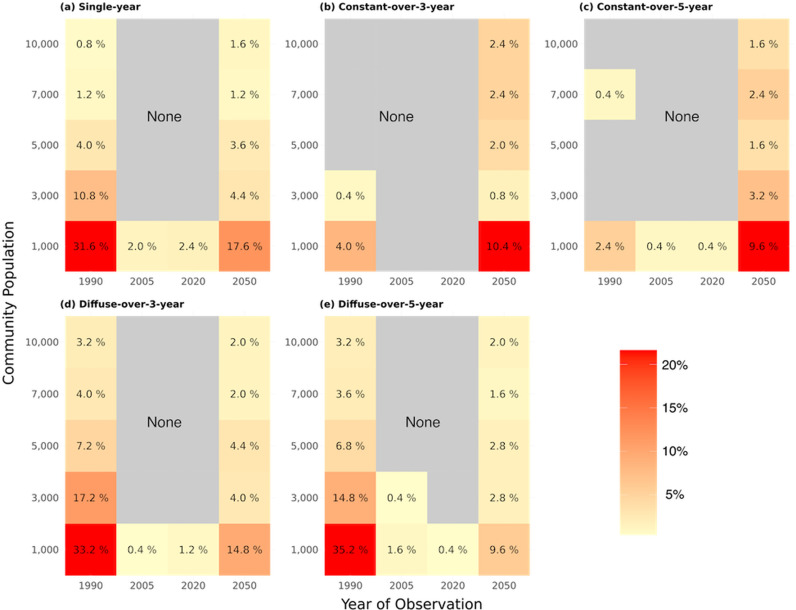
Proportion of communities identified as prevalence outliers across observation years, community population and HIV case introduction pattern. The proportion of communities identified as prevalence outliers for different HIV case introduction patterns and initial population sizes. The HIV case introduction patterns are: a: single-year (1 introduction event with p), b: constant-over-3-year (3 introduction events with p), c: constant-over-5-year (5 introduction events with p), d: diffuse-over-3-year (3 introduction events with $\frac{p}{3}$), and e: diffuse-over-5-year (5 introduction events with $\frac{p}{5}$). The columns represent the observation years (1990, 2005, 2020, 2050). The rows correspond to the initial population sizes. Each cell in the heatmap indicates the proportion of communities identified as prevalence outliers for a specific combination of HIV case introduction pattern, population size, and observation year.

Accordingly, the proportion of simulations classified as HIV prevalence outliers was highest in the early and late epidemics and lowest during the epidemic peak (Fig 3). The figure reveals that smaller communities (e.g., 1,000 individuals) consistently show a higher proportion of prevalence outlier formation across all introduction patterns, particularly in the early (1990) and late (2050) stages of the epidemic. This indicates that random fluctuations in initial seeding contribute to spatial heterogeneity in HIV prevalence, particularly in smaller communities where stochastic effects play a more pronounced role. Interestingly, during the epidemic peak (2005), the proportion of identified prevalence outliers decreased across most population sizes, suggesting that randomness plays a smaller role when HIV prevalence is high and relatively homogeneous across communities due to widespread transmission and more consistent control measures. Among the smallest communities simulated (initial population size of 1,000), which were the most prone to forming outliers, a single-year introduction of HIV cases led to 31.6% of simulations being classified as outliers in 1990, 2.0% in 2005, 2.4% in 2020, and 17.6% in 2050. For the largest communities simulated (initial population size of 10,000), a single-year introduction of HIV cases led to 0.8% of simulations being classified as outliers in 1990, no outliers observed in 2005 or 2020, and 1.6% in 2050.

The initial risk distribution of seeded individuals, as defined by the risk-stratified case importation fraction, influenced the occurrence of HIV prevalence outliers (S1 Fig). Communities where initial HIV cases occurred in higher-risk groups were more prone to becoming HIV prevalence outliers. This effect was consistent across different HIV importation patterns and initial population sizes.

### Temporal patterns of outliers

**Fig 4:**
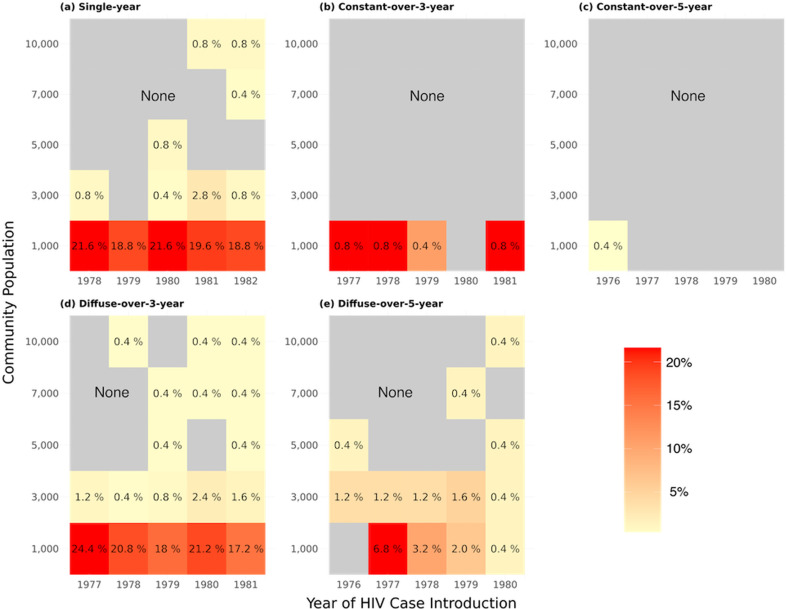
Proportion of communities identified as persistent prevalence outliers across community population and HIV case introduction pattern. The proportion of communities identified as persistent prevalence outliers for different HIV case introduction patterns and initial population sizes. The HIV case introduction patterns are: a: single-year (1 introduction event with probability p), b: constant-over-3-year (3 introduction events with p), c: constant-over-5-year (5 introduction events with p), d: diffuse-over-3-year (3 introduction events with $\frac{p}{3}$), and e: diffuse-over-5-year (5 introduction events with $\frac{p}{5}$). The columns represent the years of the first HIV introduction (1978, 1979, 1980, 1981, 1982). The rows correspond to the initial population sizes. Each cell in the heatmap indicates the proportion of communities identified as persistent prevalence outliers for a specific combination of HIV case introduction pattern, population size, and year of HIV case introduction.

The persistence of HIV prevalence outliers decreased with increasing population size (Fig 4). The columns represent the years of the first HIV introduction (ranging from 1978 to 1982), while the rows indicate various initial population sizes. Each cell in the heatmap displays the proportion of persistent prevalence outliers for a specific combination of introduction pattern, population size, and seeding year.

The results indicate that smaller populations (e.g., 1,000 individuals) are more likely to develop persistent prevalence outliers, particularly when the introduction of HIV cases occurs early (e.g., 1978) and is concentrated within a brief seeding period (e.g., single-year pattern). This trend is less pronounced in larger populations, suggesting that the impact of random fluctuations decreases as population size increases. Across the 375 scenarios modeled, the combination of a low demographic risk profile, earlier epidemic seeding (1978), small population (1,000), and brief seeding (single-year introduction pattern) was associated with the largest proportion of persistent HIV prevalence outliers, at 21.6%. While higher-risk groups were more likely to experience early outlier formation, low-risk populations exhibited greater outlier persistence due to lower transmission rates, which limited epidemic saturation and allowed stochastic effects to have a prolonged influence.

Autocorrelation analysis was consistent with persistence analysis in terms of high prevalence and factors associated with outlier status (Table 1). The highest autocorrelation value for outlier persistence was comparing the years 1990–2005 in a population of 1,000, with an autocorrelation of 42.4%. High autocorrelation was also observed for the pairs of years (2005, 2020) and (2005, 2050) in this population size, both at 23.0%. These findings underscore how smaller communities are prone to stochastic fluctuation, which can lead to the formation of persistent prevalence outliers.

**Table 1:** Autocorrelation of HIV prevalence across different time intervals and population sizes.

	Proportion of Prevalence Outlier (%)				
**Population Size**	**1990 & 2005**	**1990 & 2020**	**1990 & 2050**	**2005 & 2020**	**2005 & 2050**
1,000	42.4	30.8	31.6	32.0	32.0
3,000	22.4	0.4	8.4	0.4	6.8
5,000	12.4	0.0	4.0	0.0	4.0
7,000	10.0	0.0	2.0	0.0	1.6
10,000	4.4	0.0	2.0	0.0	1.6

The autocorrelation of prevalence is analyzed between the years 1990 and 2005, 1990 and 2020, 1990 and 2050, 2005 and 2020, as well as between 2020 and 2050, across initial populations of 1,000, 3,000, 5,000, 7,000, and 10,000 individuals. Scenarios with a low demographic risk profile and 1 seed in 1978.

### Sensitivity to alternative methods of outlier detection

To further validate the persistence of outliers, we applied Density-Based Spatial Clustering of Applications with Noise (DBSCAN) as an alternative method to detect outliers. DBSCAN identifies outliers based on local data point density, allowing for the detection of simulations with prevalence rates that deviate from the majority, independent of any pre-specified threshold. DBSCAN results revealed a higher proportion of persistent outliers in smaller populations during 2005 and 2020, aligning with the threshold-based classification. This consistency suggests that stochastic effects play a substantial role in driving persistent prevalence outliers, regardless of the detection method used.

### Sensitivity to alternative outlier definition thresholds

To further explore the robustness of our definition of outlier creation, we conducted a sensitivity analysis by varying the threshold value for defining outlier formation from 1.1 to 3 times the median HIV prevalence. When the threshold was set to 2 times the median prevalence, few outliers were detected across all simulation scenarios (S2 Fig). This observation was consistent across all initial population sizes and HIV case introduction patterns. When we further varied the persistent outlier definition threshold from 50% to 100% of the simulated time period, reducing the threshold to 50% of the time period allowed for persistent outliers to detected for the population size of 1,000, but not for larger population sizes (S3 Fig).

Using DBSCAN, a higher proportion of simulations were identified as outliers in 2005 and 2020, which may reflect the epidemic dynamics during its expansion and stabilization phases, compared to 1990 and 2050 across all population sizes. Specifically, in 2005, 6.3% of the simulations across all population sizes were classified as outliers, while in 2020, this increased to 6.8%. During these years, community-level differences in transmission and intervention uptake could have amplified random fluctuations, leading to more communities deviating from the median prevalence. In contrast, the proportion of outliers in 1990 and 2050 was lower, with 1.6% and 0.1% of simulations, respectively. Smaller population sizes showed a higher propensity for outliers. In populations of 1,000 individuals, 23.8% of simulations were classified as outliers across the years, while in populations of 10,000 individuals, the outlier proportion dropped to 4.8%. This trend was consistent across all time points.

## Discussion

This study explored the role of random fluctuations in the emergence and persistence of HIV prevalence outliers. We found that randomness can drive prevalence outlier formation, especially in smaller communities—a finding that was robust to alternative methods of outlier detection. In smaller communities, stochastic effects alone can result in the emergence and persistence of prevalence outliers, as random fluctuations in early case introductions disproportionately influence long-term prevalence trends. In contrast, larger communities typically require additional structural or behavioral factors—such as behavioral risks or gaps in prevention and treatment—for outliers to arise and persist. In smaller communities, limited sexual network connectivity and fewer external introductions can also reinforce high-prevalence states once established. Interestingly, the pattern and timing of HIV case importation—whether it occurred as an intense one-time event or diffused over several years—had minimal impact on outlier emergence and persistence. This suggests that once a community becomes a prevalence outlier due to stochastic effects, it may remain in a high-prevalence state over time, particularly in the absence of external mitigating factors. This persistence is likely driven by the long-term nature of HIV infection and the self-perpetuating cycle between high prevalence and high incidence.

The quantitative results reinforce this distinction between smaller and larger populations. In communities of at least 5,000 individuals, prevalence outliers defined as>1.5x the median prevalence were unlikely (<5%) to emerge due to randomness alone. Similarly, in communities of at least 3,000 individuals, the persistence of randomly formed prevalence outlier was rare. These findings suggest that while stochasticity plays a central role in smaller communities, larger populations require additional epidemiological mechanisms—such as risk heterogeneity, mobility patterns, or differential access to prevention and treatment services—for outliers to form and persist. Although our model was calibrated to Kisumu County, the findings are not intended to be directly generalizable to all regions of sub-Saharan Africa. Differences in epidemiology, population structure, and healthcare access across settings may influence the extent to which stochastic processes contribute to prevalence outlier formation. Therefore, caution is warranted when extrapolating these results to other regions.

These findings contribute to a broader understanding of why prevalence outliers are formed and highlight the challenges in identifying their drivers. Similar to “patchy” epidemic patterns observed in other pandemics like COVID-19, where hotspots do not align neatly with known risk factors [46–48], HIV prevalence outliers in smaller communities may arise due to inherent stochasticity rather than specific, observable predisposing factors. This pattern has been observed in rural and peri-urban areas of Africa, where neighboring communities with comparable risk behaviors still experience striking differences in HIV burden [15–17]. Such spatial heterogeneity has been documented in high-prevalence geographies such as KwaZulu-Natal [37], Eswatini [46], and Lake Victoria basin [47], suggesting that stochastic effects could play a role in shaping prevalence outliers across multiple high-burden regions. Understanding how randomness contributes to these patterns is essential for refining epidemiological models and improving targeted intervention strategies.

However, while our results emphasize the role of randomness in prevalence outlier formation, they do not entirely dismiss the role of unobserved factors. In larger and more interconnected sexual networks, random fluctuations alone may be insufficient to explain the formation of prevalence outliers, suggesting that other factors—such as differential risk behaviors [30,33,34], social norms [29], or variations in access to healthcare [49]—may play a crucial role. Further research is needed to uncover these mechanisms in larger populations.

Community size—particularly when small—is a critical factor amplifying the influence of randomness, but other elements may also intensify these effects. Population mobility, social network connectivity, and localized health behaviors could introduce more complexity [50]. Small communities with closed or less interconnected sexual networks might be particularly susceptible to random variations, leading to concentrated outbreaks.

Recognizing randomness as a potential driver of prevalence outlier formation carries important implications for public health, particularly in HIV control efforts. One of the key challenges is that prevalence outliers driven by random fluctuations are inherently difficult to detect using traditional surveillance methods, which often rely on proxy indicators, such as behavioral risk factors or the presence of high-risk subpopulations [15,51]. Common approaches such as serosurveys (e.g., population-based surveys and ANC surveillance) and geospatial modeling of case data provide valuable insights [5,10,11,52], but they often require prohibitively large sample sizes to achieve the necessary granularity over large populations. As a result, these methods may overlook prevalence outliers that arise primarily due to stochastic effects [42,43] rather than identifiable risk factors, potentially leading high-prevalence areas undetected and missing opportunities for timely intervention. Addressing this challenge may require more spatially granular surveillance strategies [5,15], particularly in smaller communities. However, achieving this level of granularity over large populations requires prohibitively large sample sizes, making it challenging to implement at scale [53]. Serosurveys could help detect emerging hotspots early, even when they arise through stochastic processes [54]. Early detection is crucial, as it can prevent communities from becoming “trapped” in a high-prevalence state, a scenario that is particularly difficult to reverse given the long-term nature of HIV infection.

Our findings have broader implications for other infectious diseases with spatial clustering, such as tuberculosis, malaria, and COVID-19. Many of these diseases exhibit patchy incidence patterns, suggesting that stochastic processes could shape their spatial dynamics alongside biological, environmental, and behavioral drivers. For example, in the case of tuberculosis (TB), the transmission dynamics in high-burden settings are often influenced by local variations in population density, healthcare access, and socioeconomic conditions [55]. Our findings indicate that in smaller, more localized populations, the random introduction and spread of infection—amplified by pre-existing conditions like poor ventilation or malnutrition—could create persistent clusters independent of these deterministic risk factors [56]. This highlights the importance of incorporating randomness into models and surveillance strategies for TB to better identify and target these clusters. In vector-borne diseases like malaria, random introduction of infected individuals or small-scale variability in vector density could lead to unpredictable hotspots [57]. Likewise, for rapidly spreading diseases like COVID-19, understanding that randomness can drive initial prevalence outlier formation underscores the need for flexible, adaptive surveillance systems capable of real-time detection [58] and response [59]. Applying adaptive surveillance strategies informed by these insights could enhance early detection and prevent widespread outbreaks by targeting resources and interventions to areas at risk of random epidemic emergence.

Overall, by highlighting the impact of randomness in the spatial dynamics of HIV, this study provides a framework for understanding how stochastic processes contribute to the emergence of prevalence outliers in HIV epidemics. Incorporating stochastic processes into epidemic models [60–62] and public health strategies will be essential for accurately predicting and managing the spatial patterns of these diseases, enabling more efficient use of resources and improving disease control and prevention efforts globally.

Future research should explore how ongoing and intermittent HIV case introductions, social network evolution, and varying access to healthcare services interact with stochastic processes to influence prevalence outlier formation in larger populations. Expanding the modeling framework to include genetic diversity in HIV strains [63], co-infections [64,65], and geographical migration patterns [37,52,66] could also provide a more comprehensive understanding of epidemic dynamics. Additionally, applying these approaches to other infectious diseases will help verify the broader applicability of these findings, ultimately improving our ability to predict, monitor, and control diverse outbreaks.

Our study has several important limitations. First, the modeling framework lacked explicit geographic demarcation of individual communities and inter-community migration. However, it did include a detailed sexual network. Social distance and geographic distance correlate, but social community sizes are ultimately impacted by multiple [64] factors including availability of transportation [15], labor migration patterns [67], social and cultural differences, and social norms around inter-cultural or inter-geographic sexual partnerships. Thus, interpretation of results through a geographic lens should be performed with caution and context-specific information.

Second, limited real-world data were available to validate our model, given that only a few HIV surveillance studies sampled participants densely enough to assess differences in HIV prevalence across small-scale communities and neighborhoods. Our findings were consistent with the magnitude of HIV prevalence differences observed in HIV surveillance sites in Africa [15–17], but given the limited number of sites that have undergone comprehensive HIV surveillance, we had insufficient data to formally validate our predictions.

Third, we focused exclusively on the initial importation of HIV cases in the early stages of the epidemic and did not consider the potential for new HIV importations occurring later in the epidemic. In reality, HIV is continually imported into communities–a process overlooked by our model and others [68–70]. However, as epidemics stabilize, the relative impact of migration-driven HIV introductions may diminish compared smaller relative to the size of the established epidemic. This pattern has been observed in other infectious disease settings where migration effects are most pronounced during early epidemic growth but play a reduced role once equilibrium is reached [71–74]. Nonetheless, even in stabilized epidemics, migration can still substantially contribute to incidence and prevalence, as highlighted in recent studies [75]. Future analysis could explore the effects of ongoing and intermittent HIV introductions throughout the epidemic.

Fourth, there is no widely-accepted quantitative threshold to define an HIV prevalence outlier. Our main analysis used an arbitrarily-chosen threshold of 1.5x the median of otherwise identical communities. However, in sensitivity analysis, we explored a wide range of alternative threshold definitions: 1.1x to 3x the median.

Finally, like all models, our model relies on assumptions in both structure and parameters. Parametrically, our model was calibrated to HIV epidemiology in Kisumu, Kenya, which may limit generalizability to other settings. Structurally, our model made numerous simplifications. For example, non-HIV STIs are modeled as a static risk rather than co-transmitting on the sexual network; [76] we modeled only heterosexual and vertical HIV transmission (the primary transmission modalities in Eastern and Southern Africa); and certain biological details such as genomic differences in HIV virulence, transmissibility, and drug resistance are not included in the model [63]. However, the model chosen in this study is among the most detailed social network models available for HIV transmission in the African context and was particularly well-suited to study HIV prevalence outlier formation. Future research could revisit prevalence outlier analyses with alternative models and explore the amplification of random fluctuations in other infectious diseases beyond HIV.

## Conclusions

Our study is the first, to our knowledge, to examine the role of random perturbations in the formation and persistence of HIV prevalence outliers. Smaller communities with relatively low rates of case importation appear to be relatively more prone to forming random HIV prevalence outliers than larger communities, with implications for HIV surveillance and for basic understanding of epidemic patterns in HIV and other infectious diseases. Recognizing that randomness can contribute to prevalence outlier formation underscores the value of adaptive public health strategies that consider stochastic processes alongside known epidemiological drivers. Integrating stochasticity into both surveillance and intervention efforts can lead to more resilient and adaptive responses, better suited to the complex and often unpredictable nature of epidemics. This shift in perspective may contribute to refining epidemic control strategies for HIV and other infectious diseases by emphasizing the importance of accounting for stochastic process.

## Materials and methods

### Model framework and calibration

We utilized the EMOD-HIV model, a previously validated and widely-used [60,77–80] agent-based HIV network transmission model developed by the Institute for Disease Modeling [61], to investigate the role of random fluctuations in the origins of HIV prevalence outliers. EMOD-HIV simulates the natural history of HIV infection, the effect of HIV treatment on health and transmissibility, and impact of HIV prevention, with care and prevention distributed according to a detailed care continuum [81].

As an agent-based model, EMOD-HIV allows for detailed representation of individual-level behaviors, relationship dynamics, and variations in sexual networks. HIV transmission is simulated on a sexual contact network, with different relationship types (in order from longest to shortest: marital, informal, transitory, and commercial) each having characteristic age-patterns of formation, duration, condom usage probability, and probability of openness to extra-relational sexual partners [82]. These elements are critical for understanding how random fluctuations in transmission can lead to varying epidemic outcomes. Additionally, EMOD-HIV simulates transmission at the level of individual coital acts, while accounting for factors such as condom use, antiretroviral therapy (ART) adherence, and sexually transmitted infections (STI) co-infections, makes it an ideal tool for exploring how small-scale random events and initial conditions can propagate to create larger spatial disparities in HIV prevalence. Vertical transmission from an infected mother to a child is also simulated, with transmission risk modified by the mother’s use of effective ART or other antiretroviral medications for prevention of mother-to-child transmission (PMTCT). By allowing different patterns of case introductions and utilizing random number seeds to generate a wide range of potential epidemic trajectories, EMOD-HIV provides a robust platform for investigating how random perturbations in sexual networks and transmission dynamics may contribute to the emergence and persistence of HIV prevalence outliers.

EMOD-HIV was previously calibrated to HIV prevalence trends in a range of settings in Africa, including the Nyanza region of western Kenya, where it was validated by predicting HIV incidence in a large cluster-randomized trial measuring HIV incidence in sixteen communities [83]. These predictions were published while the trial was blinded, and then successfully validated against HIV incidence measurements. This western Kenya model has been used in several HIV prevention modeling studies [60,77,84,85]. Likewise, Kisumu County was chosen as the illustrative example in this study due to its status as a well-documented HIV hotspot within the Nyanza region of western Kenya, an area that consistently reports some of the highest HIV prevalence rates in the country. This specific geography provides a relevant and representative setting for studying the role of randomness in prevalence outlier formation, as it combines high HIV burden with diverse community structures, varying population densities, and interconnected social networks.

The original model is subdivided into six geographic regions corresponding to the six counties of the Nyanza region: Homa Bay, Kisii, Kisumu, Migori, Nyamira, and Siaya Counties. For this study, we selected the Kisumu County geography as an illustrative example to investigate the emergence of outliers and assess whether they can arise from random fluctuations. Each simulation represents a single, independent community, meaning that no explicit spatial connections, inter-community migration, or geographic heterogeneity were included. The model focuses exclusively on within-community transmission, allowing us to isolate the role of within-community stochastic variation in shaping HIV prevalence patterns.

### Simulation scenarios

To explore the factors contributing to the formation of HIV prevalence outliers, we simulated a total of 375 HIV epidemic scenarios (Table 2), varying the population size, timing of initial HIV case introduction, and the number of initially infected individuals. Each scenario was simulated with 250 different random number seeds to evaluate how stochastic fluctuations in early transmission dynamics impact the HIV epidemic. By maintaining all other epidemiological parameters constant while varying the random seed and initial seeding patterns, we specifically assessed the impact of randomness on the emergence and persistence of prevalence outliers. The 375 scenarios represented a range of initial population sizes (1,000; 3,000; 5,000; 7,000; and 10,000 individuals), timing of the start of the epidemic (1976–1984), duration during which initial HIV cases were distributed (1–5 years), and distribution of initial cases across behavioral risk groups. We then compared how random fluctuations in the HIV epidemic affected different scenarios and simulation runs, holding all other epidemiological assumptions constant.

**Table 2:** Scenarios for HIV prevalence outlier emergence.

Population	Number of Seeds	Risk Level	Risk-Stratified Case Importation Fraction		Years of HIV Case Introduction				
1,000 3,000 5,000 7,000 10,000		High	0.0750		1978	1979	1980	1981	1982
	1	Medium	0.0180						
		Low	0.0071						
			p	$\frac{p}{3}$					
		High	0.0750	0.0250	1977	1978	1979	1980	1981
	3	Medium	0.0180	0.0060	1978	1979	1980	1981	1982
		Low	0.0071	0.0024	1979	1980	1981	1982	1983
			p	$\frac{p}{5}$	1976	1977	1978	1979	1980
		High	0.0750	0.0150	1977	1978	1979	1980	1981
	5	Medium	0.0180	0.0036	1978	1979	1980	1981	1982
		Low	0.0071	0.0014	1979	1980	1981	1982	1983
					1980	1981	1982	1983	1984

The different simulation scenarios explored in this study. *Population* refers to the initial number of individuals in each simulated community. *Number of Seeds* represents the number of years over which initial HIV cases were introduced. *Risk Level* categorizes individuals based on their likelihood of acquiring HIV: High (7.5% of individuals seeded), Medium (1.8% seeded), and Low (0.7% seeded) if seeded at once. *Risk-Stratified Case Importation Fraction* indicates the proportion of individuals within each risk group who were initially seeded with HIV cases. The value *p* represents the seeding fraction for each group, while $\frac{p}{3}$ and $\frac{p}{5}$ correspond to scenarios where case introduction was spread over three or five years, respectively. *Years of HIV Case Introduction* denotes the years when initial infections were introduced into the population.

### Patterns of HIV case introduction

In epidemiological modeling, HIV-positive cases are introduced into the simulation to initialize the modeled epidemic in a process known as “seeding”. A random number seed is a value that initializes the random number generator used in the simulation, ensuring that each run has a unique sequence of random events, such as case introductions. In this study, we conducted comparisons across identical community configurations, where the only difference between simulations was the random number seed. This approach allows us to isolate the effects of stochastic variability and assess whether observed differences in HIV prevalence outliers could arise purely due to chance. Models generally only apply seeding early in a simulation to represent a net inflow and outflow (migration) of cases [62,68–70].

**Fig 5:**
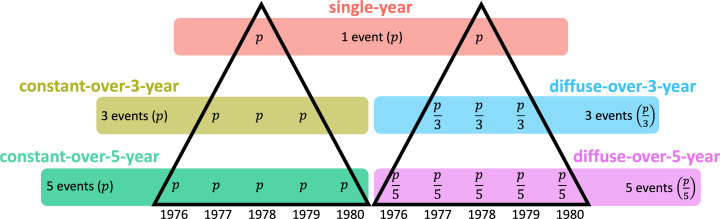
Overview of HIV case importation pattern in simulation scenarios.

We modeled five different patterns of seeding, illustrated in Figs 5 and S4, each reflecting a chosen intensity and duration of case introduction into a community. In the single-year pattern, all cases (denoted as p) are introduced within a single year (e.g., 1978). In the diffuse-over-3-year pattern, introduction of cases is spread evenly over three years (e.g., from 1978 to 1980), with $\frac{p}{3}$ cases introduced per year, totaling p over the three-year period. Similarly, in the diffuse-over-5-year pattern, introduction of cases is spread evenly over five years (e.g., from 1976 to 1980), with $\frac{p}{5}$ cases introduced per year, summing up to p. In other scenarios, we allowed the number of introduced cases to rise in proportion to the seeding duration. In the constant-over-3-year pattern, p cases are introduced each year, so that a total of 3p cases are introduced. Similarly, in the constant-over-5-year pattern, a total of 5p cases are introduced. After this initial seeding period, no further HIV case importations occur in the simulations. All subsequent HIV transmission arises solely from within-community sexual networks, without external introductions. Initial seeding was conducted within three predefined risk groups (low, medium, and high risk), with a fixed proportion of individuals in each group assigned as initial cases. Specifically, p=7.5% for the high-risk group, p=1.8% for the medium-risk group, and p=0.7% for the low-risk group. These proportions remained constant across all simulations to ensure comparability across scenarios.

### Definition of HIV prevalence outlier existence and persistence

We defined HIV prevalence outlier community both cross-sectionally (outlier existence) and longitudinally (outlier persistence) based on deviations in HIV prevalence from the median of simulated communities under identical conditions. Outlier existence was initially defined by comparing HIV prevalence of the community at a given time (e.g., 1990) to the median prevalence across other simulation runs in the ensemble, which were simulated under identical assumptions and differed only due to stochastic variation. A community was classified as an outlier if its prevalence exceeded 1.5 times the median of communities configured with identical assumption. The choice of 1.5x the median was made to strike a balance between detecting substantial deviations in prevalence and obtaining a statistically robust sample of hotspots across a range of model assumptions regarding case importation. However, we recognize that this threshold is arbitrary, and to assess the robustness of our results, we conducted sensitivity analyses using alternative thresholds ranging from 1.1x to 3x the median prevalence.

Additionally, we applied Density-Based Spatial Clustering of Applications with Noise (DBSCAN) as an alternative method to detect outliers. DBSCAN identifies outliers based on local data point density, which allows for detecting replicates with prevalence rates that deviate from other replicates, regardless of the predetermined threshold.

To evaluate outlier persistence, we performed simulations spanning the period from epidemic seeding until 2050 in monthly time-steps. Simulations were classified as persistent outliers if they met criteria for outlier existence for ≥70% of the simulated time-steps (137–149 time steps). The 70% threshold was selected to align with the time period from the center of seeding (1980) to the target of HIV epidemic control by 2030. This threshold was also varied in sensitivity analysis. DBSCAN was also applied to evaluate outlier persistence. To ensure robust detection of persistent outliers, we selected a threshold of 20 time steps. This value was chosen based on the observed distribution of outlier durations.

### Statistical analysis

We used two-sample t-tests to compare HIV prevalence across different scenarios. To understand longitudinal patterns of outlier persistence, we calculated the autocorrelation of outlier status between specific pairs of observed years. Autocorrelation was calculated to assess the temporal persistence of outliers, providing insight into whether random prevalence outlier formation tends to persist over time. Autocorrelation was defined as the proportion of simulations identified as outliers in one year (e.g., 1990) that were also identified as outliers in a different year (e.g., 2005). This process was repeated for different year pairs. P-values were calculated with a null hypothesis of no correlation between prior and subsequent outlier status at the community level. Statistical analyses were performed using R version 4.0.3.

## Supporting information

S1 Fig Impact of population size, risk-stratified case importation fraction, and HIV case introduction pattern on the proportion of persistent HIV prevalence outliers. (TIFF)

S2 Fig Sensitivity analysis of HIV prevalence outlier proportions by population size, case introduction pattern, and observation year. (TIFF)

S3 Fig Sensitivity analysis of persistent HIV prevalence outliers by case introduction pattern, seeding year, and population size (A. high risk profile, B. medium risk profile, C. low risk profile). (TIFF)

S4 Fig Overview of HIV case importation pattern in simulation scenarios. (TIFF)

S1 Table Pairwise comparisons of mean HIV prevalence in 2018 by initial population size. (XLSX)

S2 Table Pearson correlation coefficients for HIV prevalence across various year pairs. (XLSX)
