# Supplementary figures and images for: Uncovering the impact of randomness in HIV hotspot formation: A mathematical modeling study
Source: PLoS Comput Biol. 2025 Jun 16;21(6):e1013178. doi: 10.1371/journal.pcbi.1013178 (PMC12201661; doi:10.1371/journal.pcbi.1013178)

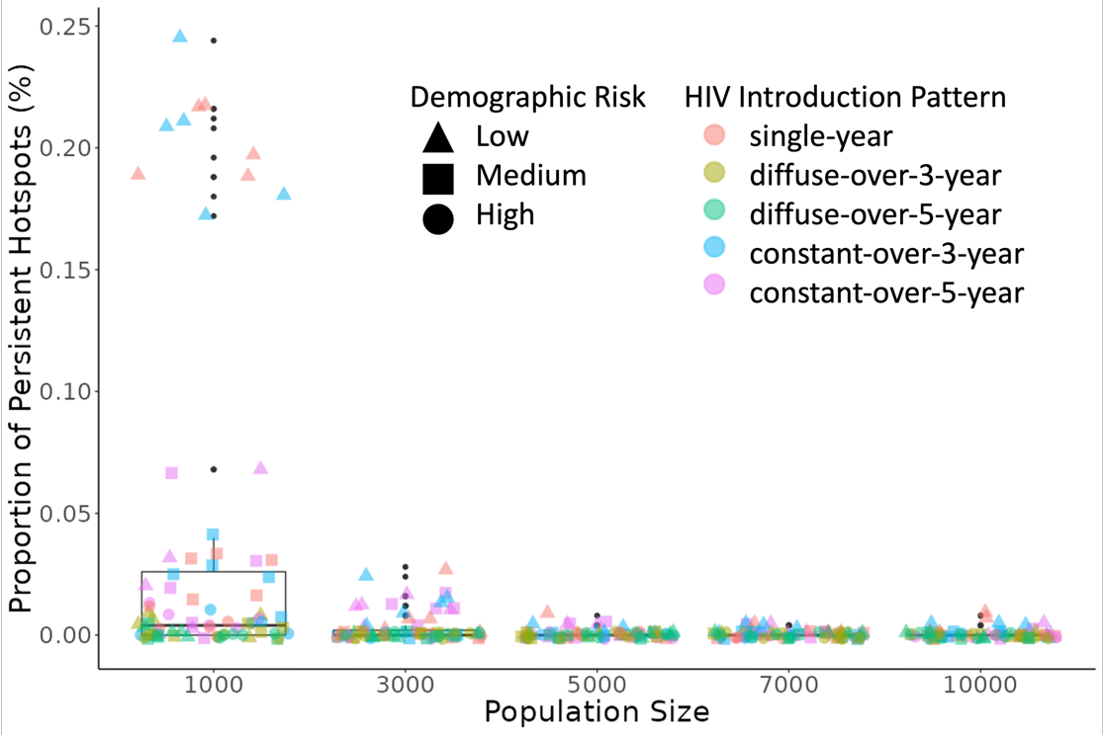

Supplement: S1 Fig — (TIFF) [file pcbi.1013178.s001.tiff]

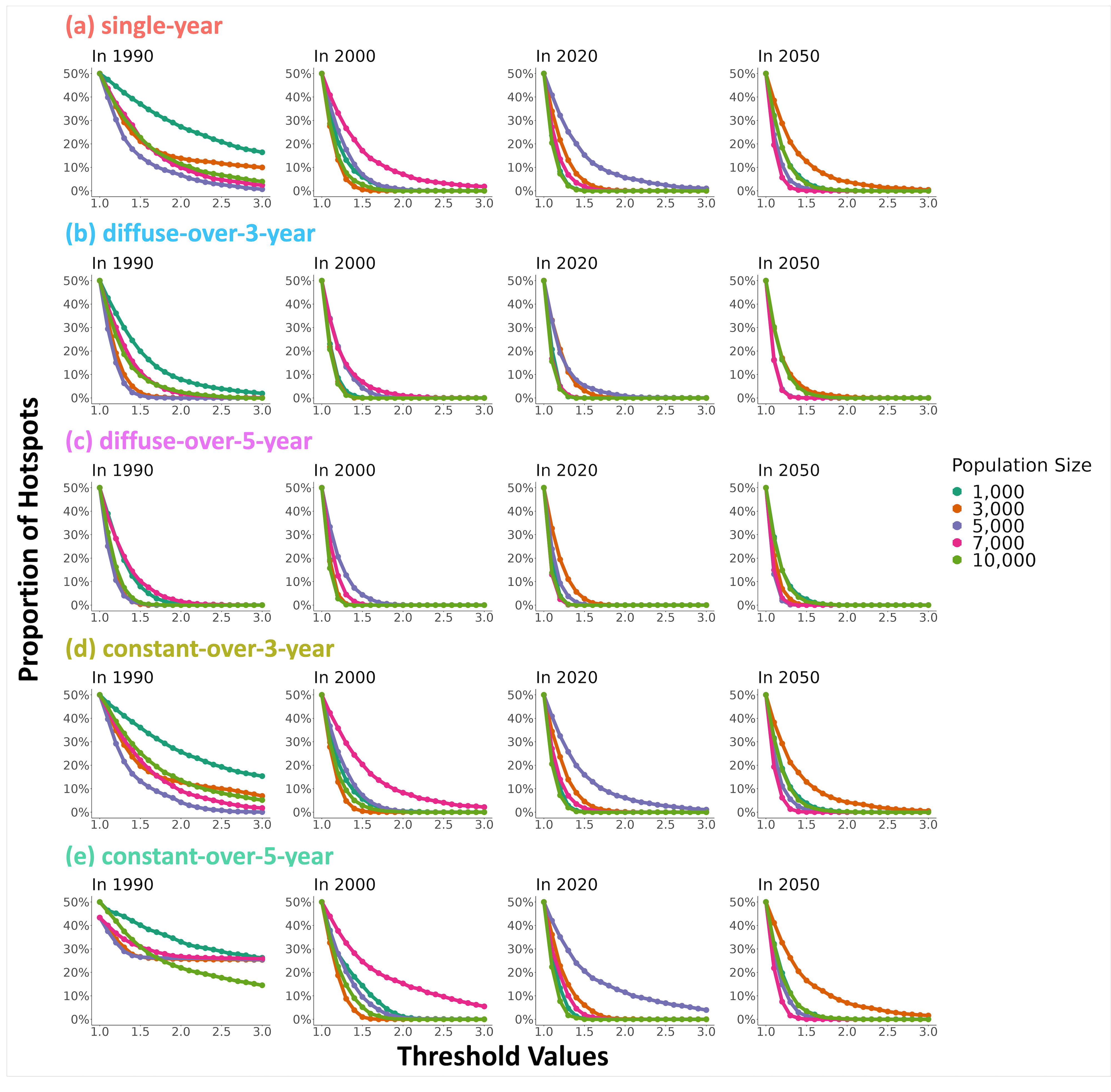

Supplement: S2 Fig — (TIFF) [file pcbi.1013178.s002.tiff]

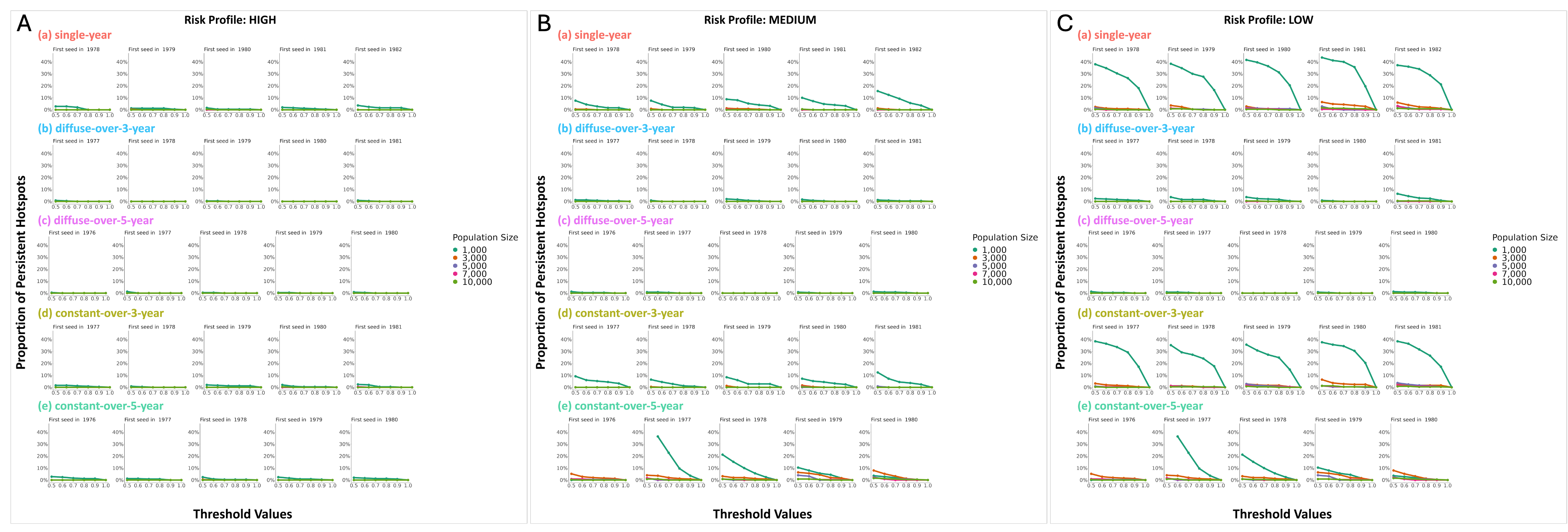

Supplement: S3 Fig — (TIFF) [file pcbi.1013178.s003.tiff]
